# Review of Clinical Features, Microbiological Spectrum, and Treatment Outcomes of Endogenous Endophthalmitis over an 8-Year Period

**DOI:** 10.1155/2012/265078

**Published:** 2012-02-23

**Authors:** Zenith H. Y. Wu, Rose P. S. Chan, Fiona O. J. Luk, David T. L. Liu, Carmen K. M. Chan, Dennis S. C. Lam, Timothy Y. Y. Lai

**Affiliations:** Department of Ophthalmology & Visual Sciences, The Chinese University of Hong Kong, Hong Kong

## Abstract

*Purpose*. To evaluate the clinical features, microbiological spectrum, and treatment outcomes of endogenous endophthalmitis. *Methods*. Retrospective review of consecutive cases with infective endogenous endophthalmitis presenting from 2000 to 2007. The main outcome measure was the visual outcome at the latest follow-up visit. Other outcome measures included microbiological investigations, anatomical and clinical outcomes. *Results*. 22 eyes of 21 patients were included, and the mean follow-up duration was 2.7 years. Eyes with fungal endogenous endophthalmitis were more likely to have visual acuity of finger counting or better at presentation compared with those with bacterial endogenous endophthalmitis (odds ratio = 15.0, *P* = 0.013). Gram-negative microorganisms accounted for 50% of infections, while fungal and gram-positive organisms accounted for 27.3% and 22.7%, respectively. Despite treatment, the visual outcome was poor in general as 10 (45.5%) eyes had no light perception at the latest follow-up visit and 6 (27.3%) eyes required enucleation or evisceration. Contrary to previous studies, fungal endogenous endophthalmitis did not appear to have better visual outcome compared with bacterial endogenous endophthalmitis. *Conclusion*. Gram-negative microorganisms were the main causative pathogens of endogenous endophthalmitis in Hong Kong. The visual prognosis of endogenous endophthalmitis is generally poor as almost 50% of eyes were blind despite treatment.

## 1. Introduction

Endophthalmitis is defined as inflammation of intraocular space secondary to intraocular infection. It can be classified into exogenous or endogenous forms. Exogenous endophthalmitis generally arises from a direct breech of external-ocular barrier as a complication of ocular trauma or intraocular surgery. Endogenous endophthalmitis is a rare form of endophthalmitis as it accounts for only 2–8% of all endophthalmitis cases [1, 2]. Endogenous endophthalmitis is caused by breeching of the blood-ocular barrier by pathogens and is spread by blood-borne route originating from infective foci, for example, endocarditis, liver abscess, urinary tract infection (UTI), and meningitis. Although the infective foci can often be identified, isolated occurrence from iatrogenic source such as dental surgery and contaminated intravenous fluids are also possible [3, 4]. A number of systemic conditions might predispose patients in developing endogenous endophthalmitis, including diabetes mellitus, cardiac diseases, underlying malignancy, immunosuppression, and intravenous drug abuse [5].

The causative organisms of endogenous endophthalmitis have been found to vary largely between different geographical locations [5–7]. In most published series, which contained both fungal and bacterial endogenous endophthalmitis, fungal infections were the most common causes [8–10]. For bacterial endogenous endophthalmitis, gram-positive organisms were more prevalent in North America and Europe, while gram-negative organisms were more commonly found in Asia. Prompt diagnosis and treatment of endogenous endophthalmitis are important as the final visual outcome of endogenous endophthalmitis is potentially devastating. Studies have suggested that endogenous endophthalmitis due to fungal infection, in particular *Candida *species, were more likely to result in better visual outcome [8–10]. The aim of this study is to evaluate the clinical features, causative organisms, infective sources, and outcomes of endogenous endophthalmitis in three tertiary eye centers in Hong Kong over an 8-year period. We also aimed to assess whether the type of infection would influence the visual outcome by comparing eyes with bacterial endogenous endophthalmitis versus those with fungal endogenous endophthalmitis.

## 2. Methods

This was a retrospective study of consecutive cases of infective endogenous endophthalmitis in three hospitals including Hong Kong Eye Hospital, Queen Elizabeth Hospital, and Prince of Wales Hospital in Hong Kong. Inclusion criteria included diagnosis of infective endogenous endophthalmitis proven by blood culture from 2000 to 2007. Exclusion criteria included patients with ocular surgery within one year of presentation, those with history of ocular trauma, and delayed onset of exogenous endophthalmitis. The clinical information studied included age and gender, preexisting ocular and medical conditions, presenting ocular features, infective foci and microbiological results, treatment, final visual outcome, and survival. The study was performed in accordance with the declaration of Helsinki and was approved by an institutional review board.

 All data were entered into a spreadsheet program (Microsoft Excel for Mac 2011, Microsoft Inc., Redmond, WA, USA) and analyzed using a statistical software (StatPlus:mac 2009, AnalystSoft Inc., Vancouver, BC, Canada). The main outcome measure of the study was the visual outcome at the latest follow-up visit, in particular, the proportion of eyes with vision of finger counting (FC) or better. Eyes which had been enucleated or eviscerated were assigned to have vision of no light perception (NLP). Other outcome measures included findings of microbiological investigations and anatomical outcome. Categorical variables were compared using the chi-square test, and odds ratios were calculated. Based on the study by Connell et al. [9], we hypothesized that eyes with fungal endogenous endophthalmitis will have a better visual prognosis, with 90% of eyes having vision of FC or better, compared with 50% of eyes for bacterial endogenous endophthalmitis. With an alpha of 5%, our sample size of 16 eyes with bacterial endogenous endophthalmitis and 6 eyes with fungal infection will have a 54% power to detect a significant difference between the two groups. A *P* value of <0.05 was considered as statistically significant.

## 3. Results

### 3.1. Clinical Characteristics

A total of 22 eyes of 21 patients (one patient with bilateral involvement) was identified to have endogenous endophthalmitis during the study period (Table 1). The mean ± standard deviation (SD) age of the patients at presentation was 61.8 ± 13.9 years (range: 22 to 89 years). There were 11 (52.3%) males. The mean follow-up duration of the patients was 2.7 ± 2.2 years (range: 1 month to 6.3 years). Twenty (95.2%) of the 21 patients presented within 30 days of the onset of ocular symptoms such as blurring of vision, eye redness, or eye pain, with only one patient presenting 90 days after the onset of ocular symptoms. The median and mean duration from the onset of ocular symptoms to presentation was 2 and 12 days, respectively (range: 1 to 90 days). Preexisting medical conditions that predisposed the development of endogenous endophthalmitis included diabetes mellitus (11 eyes, 50%), urinary tract infection (8 eyes, 36.4%), septicemia (7 eyes, 31.8%), recent general surgery (5 eyes, 22.7%), liver abscess (4 eyes, 18.2%), and malignancy (4 eyes, 18.2%). Two subjects (9%) had undergone renal transplant and were on systemic immunosuppressive therapy. Other medical conditions included indwelling catheter, intravenous drug use, pneumonia, and soft tissue abscess in one case each.

All eyes at presentation were found to have inflammation involving the anterior chamber with cells of 1+ or more. Other common ocular clinical findings included vitritis (19 eyes, 86.4%), hypopyon (13 eyes, 54.5%), conjunctival chemosis (10 eyes, 45.5%), posterior synechiae (6 eyes, 27.7%), and keratic precipitates (6 eyes, 27.7%). At presentation, eight (36.4%) eyes had vision of FC or better and the remaining had visual acuity of hand motion (HM) or worse. Two eyes were already NLP at presentation. Eyes that had endogenous endophthalmitis due to fungal infection were more likely to have vision of FC or better compared with eyes that had endogenous endophthalmitis due to bacterial infection (83.3% versus 25% resp., odds ratio = 15.0, chi-square test, *P* = 0.013).

### 3.2. Microbiology Results

Following the onset of endogenous endophthalmitis, all patients had diagnostic tapping for ocular specimen for microbiological investigations, including vitreous tap in 15 (68.2%) eyes and aqueous tap in 10 (45.5%) eyes. All diagnostic taps were performed at the time of initial presentation with ocular symptoms and prior to commencement of intravitreal antimicrobial therapy. Four samples from the vitreous tap and two samples from the aqueous tap were found to be culture positive. Blood cultures were also performed in the patients and were positive in all (100%) cases. Gram-negative organisms were found to be the causative microorganism in 11 eyes (50%), gram-positive organisms in 5 eyes (22.7%), and fungus in 6 eyes (27.3%) (Table 2). The most common microorganisms were *Klebsiella pneumoniae *(8 cases) followed by *Candida sp.* (5 cases). Infective foci could be identified in 18 patients (85.7%). The most common primary focus was urinary tract (7 eyes, 31.8%), followed by liver (4 eyes, 18.2%) (Table 1).

### 3.3. Treatment and Outcomes

Topical antibiotics (17 eyes), intravitreal antibiotics (16 eyes), and systemic antibiotics (17 eyes) were used in the treatment of bacterial endogenous endophthalmitis. Antifungal drugs, given as intravitreal injection (5 eyes) and/or systemically (6 eyes), were used in cases with fungal endogenous endophthalmitis. Topical corticosteroid treatments were also used in 9 selected cases to reduce the extent of ocular inflammation. Systemic corticosteroid was also used in one patient to control the severe intraocular inflammation associated with neovascularization of the iris. Pars plana vitrectomy (PPV) was performed in 4 (18.2%) eyes following the development of endogenous endophthalmitis, and 6 (27.3%) eyes were enucleated or eviscerated due to uncontrollable infection with NLP vision. There was a suggestive trend of having more enucleation or evisceration in eyes that had bacterial endogenous endophthalmitis (6 eyes, 37.5%) compared with fungal endogenous endophthalmitis (0 eye, 0%) but the difference failed to reach the level of statistical significance (chi-square test, *P* = 0.07).

Visual outcomes at the latest follow-up were generally poor. Ten (45.5%) eyes had VA of NLP, with five eyes having final VA between 20/400 and 20/200. There was no significant difference in the proportion of eyes having vision of FC or better at the latest follow-up between bacterial and fungal cases (25% versus 16.7%, *P* = 0.68). Eyes with more acute presentation (within 48 hours of the onset of symptoms) were less likely to have a final vision of FC or better compared with those who presented later (7.7% versus 44.4%, odds ratio = 0.10, *P* = 0.041). The final visual acuity of one patient was not available as the patient died 1 month after presentation of endogenous endophthalmitis. There were 3 (14.3%) deaths during the follow-up period, two of which were related to systemic sepsis and the third death was due to disseminated nasopharyngeal carcinoma with multiple metastases.

## 4. Discussion

Endogenous endophthalmitis is a rare form of endophthalmitis which occurs when pathogen crosses the blood-ocular barrier and causes intraocular infection. Diagnosis of endogenous endophthalmitis is mainly based on clinical findings, and empirical treatment is usually administered while waiting for the microbiological investigations results from blood or intraocular specimens. Endogenous endophthalmitis is commonly associated with systemic conditions that can cause a relative immunocompromised state [5]. In a major review of 267 cases of bacterial endogenous endophthalmitis by Jackson et al. [6], 56% of patients had an underlying condition that increased the risk of infections. In our current study, 90.9% of patients had one or more identifiable preexisting predisposing condition and the commonest systemic condition found was diabetes mellitus (50%). Other coexisting potential risk factors included UTI (36.4%), septicemia (31.8%), liver abscess (18.2%), malignancy (18.2%) and renal transplant (9.1%) with use of immunosuppressive therapy. Similar results can also be found in the literature as studies have demonstrated that up to 42% of endogenous endophthalmitis patients had underlying diabetes mellitus [6, 11]. Connell et al. [9] also reported that 14.2% with bacterial infection had coexisting malignancy.

In our series, an infective focus was identified in most of the patients with endogenous endophthalmitis. The most common source was urinary tract in 36.4%, followed by liver abscess in 18.2% and soft tissue abscess and pneumonia in 4.5% each. Chung et al. [12] reported in their series of Korean patients that 22.2% had pneumonia and 16.7% had liver abscess, while Wong et al. reported in a Singapore cohort that 48% of bacteremia arose from the hepatobiliary tract [7]. Intravenous drug use (IVDU) was reported to be another risk factor more commonly in the West. Connell et al. [9] reported a figure of 48.1% of fungal infection cases that had IVDU, while Leibovitch et al. [11] showed that that 23.1% were IVDU and all of them suffered from fungal infection. In our series, only 1 case (4.5%) of IVDU was identified but the patient had *Staph. aureus *infection rather than fungal infection.

The overall culture positive rate for intraocular specimen in our series was 24%. This relatively low positive rate revealed the difficulty of making a microbiological diagnosis in endogenous endophthalmitis. In order to improve the sensitivity and the speed of diagnosis, the use of polymerase chain reaction (PCR) technique for diagnosing endogenous endophthalmitis has been advocated [13]. However, the technique is not without disadvantages as there is a false-positive rate of 5% due to sample contamination and antibiotics sensitivity cannot be determined with PCR.

Most of the published series that have included both fungal and bacterial endogenous endophthalmitis showed that fungal endogenous endophthalmitis was more common compared with bacterial cause [8–11]. In our current series however, we found that bacterial endogenous endophthalmitis was more common. In particular, gram-negative bacteria accounted for 50% of all causative organisms. The commonest species found was *Klebsiella pneumoniae*, which was present in 8 eyes (36.3%). Fungi accounted for 27.6% of cases, with *Candida sp. *(83.3%) being the most common fungus identified. Gram-positive bacteria were found in only 22.7%. These results were different compared with those reported in North America and Europe, where the majority of organisms found were fungal or gram-positive bacteria. There has been a rising trend of gram-negative organisms causing endogenous endophthalmitis recently, especially in the South East Asia [7, 10, 12, 14–16]. The exact cause for the higher proportion of cases with gram-negative endogenous endophthalmitis is unknown but might be due to the higher prevalence of hepatobiliary infections in Asian countries [12, 17, 18].

Endogenous endophthalmitis associated with *Klebsiella *sp. is well documented to be associated with a poorer prognosis [19, 20]. In our series, 50% eyes requiring enucleation or evisceration were infected with *Klebsiella *sp. 62.5% *Klebsiella* infection coexisted with diabetes mellitus and 50% of them had liver abscess. In a 20-year review, Ang et al. [21] concluded that, in endogenous *Klebsiella *endophthalmitis, presence of hypopyon and unilateral involvement are associated with poorer prognosis. Similar association of worse prognosis in eyes with hypopyon was also observed in our patients with *Klebsiella* endogenous endophthalmitis. Six (75%) of eight eyes with *Klebsiella *endogenous endophthalmitis had hypopyon at presentation and all ended having NLP vision with 50% requiring evisceration. For the two (75%) eyes without hypopyon, the VA at presentation was HM and FC, respectively, but the final VA of these two cases both improved to 20/100 after prompt treatment.

Treatment modality for bacterial endogenous endophthalmitis employed in our series included intravitreal and systemic antibiotics and antifungal as well as surgical treatments like PPV. Intravitreal antibiotics are generally essential in treating bacterial endogenous endophthalmitis as most topical or systemic antibiotics do not reach sufficient therapeutic level in the vitreous [6]. The use of intravitreal antibiotics has been reported to reduce the chance of evisceration or enucleation but it did not significantly improve the visual prognosis [14]. PPV is another important treatment option as previous study has demonstrated that PPV can result in an 85% anatomical success rate with 80% retained a vision of FC or better after surgery [14]. In addition, the chance of requiring enucleation or evisceration was also reduced after PPV.

Despite treatment, the visual outcome of our series was generally poor. Only 22.7% of eyes had visual improvement at the latest follow-up. Although previous studies suggested that fungal endogenous endophthalmitis was associated with better vision compared with bacterial endogenous endophthalmitis [8–10], our findings showed no significant difference in the proportion of eyes with FC or better vision at the latest follow-up. Moreover, five of the six eyes with fungal endogenous endophthalmitis had NLP vision. This highlights the need to have better therapeutic agents for intraocular fungal infections.

 In conclusion, endogenous endophthalmitis is an uncommon but visually devastating condition. In contrast with previous studies, our series showed that bacterial causes in particular gram-negative bacteria were more common than fungal causes of endogenous endophthalmitis. Despite recent advances in the medical and surgical treatment of postoperative exogenous endophthalmitis, there appeared to be little improvement in the clinical outcome of endogenous endophthalmitis in the recent years. A higher index of suspicion with early diagnosis with new molecular techniques and aggressive treatment might hopefully improve the prognosis in the future.

## Figures and Tables

**Table 1:** Clinical features of 22 eyes of 21 patients with endogenous endophthalmitis.

Patient no.	Gender	Age at presentation	Infective focus	VA at presentation	VA at latest visit	Microorganism
1	Male	38	Liver	HM	NLP	*Klebsiella pneumoniae*
2	Male	71	Liver	NLP	Eviscerated	*Klebsiella pneumoniae*
3	Female	43	Nasopharynx	FC	Eviscerated	*Staph. aureus + Strep. pneumoniae*
4	Female	58	Liver	FC	20/200	*Klebsiella pneumoniae*
5	Female	75	Urinary tract	HM	NLP	*Escherichia coli*
6	Male	89	Pulmonary	20/1200	Eviscerated	*Strep. pneumoniae *
7	Male	51	Central nervous system	FC	N/A	*Candida sp.*
8	Male	42	Brain	FC	NLP	*Candida sp.*
9	Male	41	Liver	LP	NLP	*Klebsiella pneumoniae*
10	Female	67	Necrotizing fasciitis	HM	NLP	*Strep. Pyogenes*
11	Male	70	Nil	20/1200	NLP	*Aspergillus sp.*
12	Male	55	Intravenous drug	HM	20/400	*Staph. Aureus*
13	Female	75	Urinary tract	LP	NLP	*Klebsiella pneumonia*
14a	Female	60	Urinary tract	FC	20/320	*Candida sp.*
14b				20/800	NLP	*Candida sp.*
15	Female	65	Nil	LP	Eviscerated	*Klebsiella pneumonia*
16	Female	58	Urinary tract	HM	Eviscerated	*Escherichia coli*
17	Male	65	Urinary tract	HM	NLP	*Candida sp.*
18	Male	76	Nil	NLP	Eviscerated	*Klebsiella pneumoniae*
19	Female	58	Knee	HM	NLP	*Strep. agalactiae*
20	Female	86	Urinary tract	HM	20/100	*Klebsiella pneumonia*
21	Male	56	Back abscess	20/400	20/200	*Enterobacter sp.*

FC: finger counting; HM: hand motion; LP: light perception; NLP: no light perception.

**Table 2:** Microbiological results of the 22 eyes with endogenous endophthalmitis.

	No. of eyes	Percentage
Gram-positive	5	
* Staph. aureus*	1	20.0%
* Staph. aureus *+ *Strep. sp. *	1	20.0%
* Strep. pyogenes*	1	20.0%
* Strep. agalactiae*	1	20.0%
* Strep. pneumoniae*	1	20.0%
Gram-negative	11	
* Escherichia coli*	2	18.2%
* Enterobacter sp*.	1	9.1%
* Klebsiella pneumoniae*	8	72.7%
Fungal	6	
* Aspergillus sp.*	1	16.7%
* Candida sp.*	5	83.3%
